# Do mycorrhizal network benefits to survival and growth of interior Douglas-fir seedlings increase with soil moisture stress?

**DOI:** 10.1002/ece3.24

**Published:** 2011-11

**Authors:** Marcus A Bingham, Suzanne W Simard

**Affiliations:** Department of Forest Sciences, University of British ColumbiaVancouver, BC, V6T 1Z4, Canada

**Keywords:** *Pseudotsuga menziesii*var.*glauca*(interior Douglas-fir), ectomycorrhizal network, drought, ecophysiology, CO_2_, climate change, competition, facilitation, stress-gradient hypothesis, plant water relations

## Abstract

Facilitation of tree establishment by ectomycorrhizal (EM) networks (MNs) may become increasingly important as drought stress increases with climate change in some forested regions of North America. The objective of this study was to determine (1) whether temperature, CO_2_ concentration ([CO_2_]), soil moisture, and MNs interact to affect plant establishment success, such that MNs facilitate establishment when plants are the most water stressed, and (2) whether transfer of C and water between plants through MNs plays a role in this. We established interior Douglas-fir (*Pseudotsuga menziesii*var.*glauca*) seedlings in root boxes with and without the potential to form MNs with nearby conspecific seedlings that had consistent access to water via their taproots. We varied temperature, [CO_2_], and soil moisture in growth chambers. Douglas-fir seedling survival increased when the potential existed to form an MN. Growth increased with MN potential under the driest soil conditions, but decreased with temperature at 800 ppm [CO_2_]. Transfer of ^13^C to receiver seedlings was unaffected by potential to form an MN with donor seedlings, but deuterated water (D_2_O) transfer increased with MN potential under ambient [CO_2_]. Chlorophyll fluorescence was reduced when seedlings had the potential to form an MN under high [CO_2_] and cool temperatures. We conclude that Douglas-fir seedling establishment in laboratory conditions is facilitated by MN potential where Douglas-fir seedlings have consistent access to water. Moreover, this facilitation appears to increase as water stress potential increases and water transfer via networks may play a role in this. These results suggest that conservation of MN potential may be important to forest regeneration where drought stress increases with climate change.

## Introduction

Ectomycorrhizal networks (MNs) have been shown to affect seedling water budgets, but the precise nature and mechanisms underlying these effects are ambiguous (Warren et al. 2008). Large trees have been shown to play a role in redistributing water to interior Douglas-fir (*Pseudotsuga menziesii*var.*glauca*) seedlings establishing nearby, and that this is influenced by the presence of MNs (Schoonmaker et al. 2007). Similarly, oak (*Quercus agrifolia*) seedlings with access to water via their taproots have been shown to transfer some of that water to their ectomycorrhizal (EM) fungal symbionts, maintaining the integrity of the MN during drought (Querejeta et al. 2003). Thus, the potential exists for the EM fungi to distribute water among plants through an interconnecting mycelial network. Egerton–Warburton et al. (2007) also found that water was transferred between oak seedlings via MNs when one seedling was able to access water while the other was maintained in a water-deficient condition. However, this study did not assess effects on seedling survival and growth, as seedlings were exposed to drought conditions for only 12 days. Sack (2004) found a median survival time of 50 days under extreme drought for tree seedlings with a high drought tolerance, so the length of a growing season is likely ideal for assessing single-year drought effects.

MNs may also increasingly facilitate seedling establishment under increasing drought, a condition commonly expected in dry temperate forests of North America with climate change (Spittlehouse 2008). These networks, most fundamentally, inoculate the seedling and allow it to tap into an extensive preestablished EM mycelium much more rapidly than would occur via spores (Nara 2006). However, it has also been shown that plant-to-plant C transfer is facilitated by MNs along source-sink gradients, in addition to hydraulic redistribution; thus, the benefits may go beyond simple increased mycorrhizal colonization (Simard et al. 1997a; Querejeta et al. 2003). Yet, it remains to be seen whether plant-to-plant resource transfer via MNs actually influences the fitness of establishing plants.

Furthermore, water relations in plants are mediated by availability of CO_2_, which itself is a resource needed for photosynthetic assimilation (Lambers et al. 1998). As the rate of assimilation increases in response to increasing photosynthetically active radiation (PAR), intercellular CO_2_ is consumed more rapidly, and thus the plant must open its stomata more widely to replace this CO_2_. In contrast, the higher the atmospheric partial pressure of CO_2_ (*p*CO_2_), the less the plant must open its stomata to maintain a particular rate of assimilation. This reduced stomatal conductance also results in a reduced transpiration rate, and as a result, soil water is depleted less rapidly under high CO_2_. The resource acquisition ratio of C and water to other resources is kept higher for the plant. Thus,*p*CO_2_ should affect both C and water transfer between plants through MNs.

Although results have been variable, it has been experimentally shown that*p*CO_2_ affects plant growth in ecosystems (Mooney et al. 1999; Norby and Luo 2004). The*p*CO_2_ has been shown to affect transpiration, N allocation, and photosynthetic rate of Douglas-fir seedlings and saplings in a series of studies utilizing sunlit, environmentally controlled growth chambers (Lewis et al. 2002, 2004; Tingey et al. 2003). All of these physiological processes are related to C flux within the plant, and between the plant and its environment (Mooney et al. 1999). Over a 21-month growing period, these studies showed that increases in*p*CO_2_ resulted in decreased transpiration, increased instantaneous transpiration efficiency, increased foliar C/N ratio, and declining net photosynthetic rates of seedlings. Despite this photosynthetic acclimatization, net photosynthesis was still enhanced by increased CO_2_ concentration ([CO_2_]) after 21 months (Lewis et al. 2004), probably due to inhibition of photorespiration and increased RuBP regeneration mediated by the electron transport chain. While transpiration declined with increasing [CO_2_] at constant temperature, it increased with [CO_2_] (179 µmol mol^−1^ above ambient) and temperature (3.5°C above ambient), suggesting that increasing [CO_2_] will not be sufficient to counteract water deficiency in Douglas-fir seedlings in response to rising temperatures in southern British Columbia (BC) (Lewis et al. 2002).

Using the same growth chamber facilities as Lewis and group, Rygiewicz et al. (2000) assessed the 4-year response of Douglas-fir EM fungal community structure to increasing [CO_2_] and temperature, using native, low-N forest soils. During the first 2 years, EM morphotype richness increased with [CO_2_], but then plateaued for the final 2 years. Morphotype richness also increased with temperature. Greater EM fungal richness increases the probability that fungal species favorable for forming EM networks will be present, possibly increasing the importance of established parent trees to facilitation of seedling establishment in the field (Eriksson 2000; Dickie et al. 2002, 2005; Jones et al. 2003; Simard and Durall 2004; Dickie and Reich 2005).

Where water deficiency limits plant growth, any environmental factor that influences the efficiency of plant acquisition of water and/or C will help to alleviate this stress (Lambers et al. 1998). While increasing temperature-induced drought is predicted for the grassland-forest ecotones in the southern interior of BC (Hamann and Wang 2005), rising*p*CO_2_ may also affect plant and soil microbial physiological processes (Hoorens et al. 2003). Plant and regional water budgets are expected to shift, not only in response to changing precipitation and temperature regimes, but also to changes in the atmospheric*p*CO_2_. A better understanding of how MN facilitation of seedling recruitment may change with time among climatic regions is needed to forecast regeneration patterns among forest types with changing climatic conditions.

The main objective of our study was to determine whether*p*CO_2_ or temperature modulates the effect of soil moisture on potential MN facilitation of interior Douglas-fir seedling survival and growth, and whether C and water transfer play a role in this. To test*p*CO_2_ and temperature effects on the interaction among MNs and soil moisture, four growth chambers were utilized to vary*p*CO_2_ and temperature, thus simulating predictions for climate change within the range of interior Douglas-fir in BC (Spittlehouse 2008). We established receiver seedlings in root boxes with and without the potential to form MNs with larger nearby conspecific seedlings that had consistent access to water via their taproots. To test the hypothesis that water deficiency enhances facilitation by MNs, we made three predictions: receiver seedlings associated with an MN will display (1) greater survival and (2) greater growth where seedlings are growing in the lowest soil moisture, highest temperature, and lowest*p*CO_2_ conditions; and (3) C and water should transfer from the large seedling having access to a consistent water supply, to the small seedling unable to access this water, and this transfer should be greatest when the small seedling is the most water deficient.

## Materials and Methods

### Experimental design and treatments

Four growth chambers at the University of British Columbia were used. Within each growth chamber, the entire platform area at the base of each growth chamber was covered with a water reservoir we constructed from templast (corrugated plastic) and silicone. The reservoirs were filled with water and refilled to capacity at every watering.

**Figure 1:**
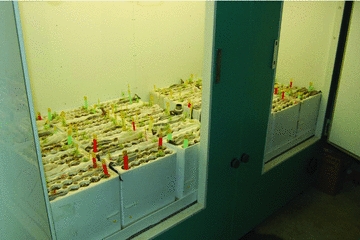
Growth chamber with single temperature regime and [CO_2_] containing mesh treatment (3 levels) and soil moisture regime (3 levels) combinations nested within. Douglas-fir seedlings can be seen growing in the containers.

Using these growth chambers, we conducted an experiment with a nested 3 × 3 × 2 × 2 factorial design, where mesh treatment (three levels) and soil moisture regime (three levels) were nested within temperature regime (two levels) and CO_2_ regime (two levels), which were applied uniquely to each growth chamber (Fig. 1). Because we had access to only four growth chambers (two retrofitted for*p*CO_2_ and the other two not) at a time, one full replicate of the*p*CO_2_× temperature treatment combinations was started in October of 2007 (run 1), and the second replicate was started in October of 2008 (run 2), after seedlings had established under uniform conditions. Within each*p*CO_2_× temperature treatment combination (2*p*CO_2_ regimes × 2 temperature regimes × 2 runs), the soil moisture × temperature treatments were replicated 10 times (3 soil moisture regimes × 3 mesh treatments × 10 replications = 90 units within each*p*CO_2_× temperature treatment; 360 units per run; 720 units for the whole experiment). Additional seedlings were planted in each treatment for natural abundance determinations.

**Figure 2:**
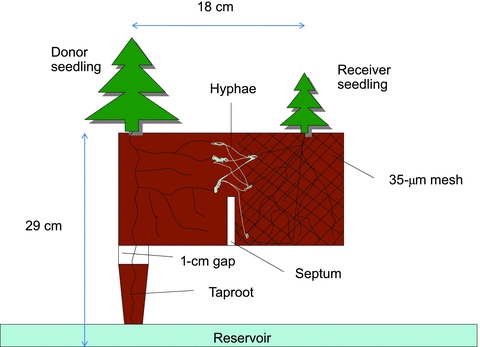
Conceptual representation of the general arrangement for donor and receiver seedlings in each root box (not to scale). In this example, the 35-µm mesh treatment has been applied to the receiver seedling.

The basic experimental unit was the root box (Fig. 2). In each root box, two stratified interior Douglas-fir seeds were planted, one near each end of the rooting compartment (after Simard et al. 1997b). One was able to access reservoir water via a taproot partition (donor seedling) (after Querejeta et al. 2003), whereas the other was not (receiver seedling). Access to an MN was controlled by lining the receiver seedling compartment with a mesh bag made of sturdy plain-weave nylon (Plastok, Birkenhead, UK). There were three “mesh” treatments: (1) no mesh, where receiver seedlings were planted directly into soil and thus could form hyphal and rhizomorph MNs with donor seedlings, and their roots were free to intermingle; (2) 35-µm mesh, where receiver hyphae could access the root systems of donor seedlings, and thus form hyphal MNs, but roots could not (after Teste and Simard 2008; Johnson et al. 2001), and (3) 0.5-µm mesh, where most hyphae and all roots of receiver seedlings were restricted from accessing donor seedlings (see Teste et al. 2006). In the no mesh treatment, receiver roots and hyphae were free to grow into the donor half of the compartment, while in the 35-µm mesh treatment hyphae were free to grow across, thus the potential volume of soil accessed for nutrient uptake increased with the potential for MN formation.

All seed originated from the Shuswap-Adams seed-planning zone (seedlot # FDI 48507). Donor seedlings were planted 1 month prior to planting of receiver seedlings, and their taproots were allowed to access reservoir water via the taproot partition to simulate a tree in the field accessing water from depths beyond the rhizosphere of an establishing seedling. To facilitate use of limited space, three root boxes (i.e., three experimental units), each randomly assigned a mesh treatment, were placed in a common container. Each container was randomly assigned one soil moisture treatment (6%, 9%, or 12%, see below) for all three experimental units.

Two temperature (warm, cool) and two*p*CO_2_ regimes (elevated, ambient) were applied to the growth chambers. In the warm temperature regime, the high temperature was 19°C and the low temperature was 16°C. In the cool temperature regime, the high temperature was 16°C and the low temperature was 13°C. These temperature regimes were chosen as a compromise between typical growing temperatures within the geographical range of interior Douglas-fir in interior BC, and in consideration of growth chamber energy consumption of cooling and refrigeration motor stress. The CO_2_-retrofited chambers were set for 800 ppm CO_2_ (δ^13^C ≈–44‰), while the nonretrofitted chambers were vented to allow equilibration with ambient levels (average measured [CO_2_] during the photoperiod for both trials was 420 ppm). PAR (400–700 nm) was measured once per month in each growth chamber using a Sunfleck PAR Ceptometer (Model SF-80, Decagon Devices, Pullman, WA) to check for drifting and adjust light height accordingly. The target PAR was 440-µmol photons m^−2^ s^−1^. The photoperiod was 16 hours (approximation of June Solstice in southern BC) for the duration of the experiment to maximize rates of growth and mortality, as well as to avoid dormancy.

### Soil

Soil for the root boxes was collected at a single representative location from nine sites across a climatic precipitation gradient in southern interior BC (Bingham and Simard, 2011), and thoroughly mixed in equal parts to proportionately capture mineral soil clast size, mineral soil composition, and fungal inocula from the full range of locations. Soil at the sites had already been mixed during site preparation, and thus was comprised of a mixture of organic and mineral horizons. The soil was collected to a depth of 32 cm and placed in coolers for transport. The soil was transferred to University of British Columbia (UBC) within 1 week of collection, and then it was immediately sieved to 4 mm, homogenized, and mixed with perlite (9:1 by volume). The root boxes were filled with soil, but a 1-cm gap was left between the soil at the bottom of a seedling compartment and the surface of the soil within the taproot partition; this was accomplished via a stricture at the hole leading from the seedling compartment to the taproot partition (Fig. 2). The gap ensured that reservoir water did not move into the seedling compartment via soil capillary action. Target average volumetric soil water contents for the three soil moisture treatments were 6%, 9%, and 12%; these levels were chosen based on growing season averages across a climatic moisture gradient within the range of interior Douglas-fir. Containers were weighed three times per week, and watered to field capacity when their weight dropped to a target level below field capacity, thus simulating naturally occurring wetting and drying cycles that fluctuated about the target soil moisture content through time.

### Pulse labeling

Prior to labeling, a subset of the extra seedlings was randomly selected for isotope analysis to determine natural abundance. Donor seedlings were randomly selected for pulse labeling with ^13^CO_2_. Donor seedlings were sealed in 0.5-L ***Tedlar*®** gas sampling bags (Cel Scientific Corp., Santa Fe Springs, CA) with septa and valves. Gas labeling bags were inflated with 100 mL of ambient air using a 0.5-L gas-tight super syringe (Hamilton, Reno, NV). One milliliter of ^13^CO_2_ (99.9%; Cambridge Isotope Labs, Andover, MA was then injected into the bag using a 1-mL gas-tight syringe (Hamilton, Reno, NV), and the donor seedling allowed to photosynthesize within the bag for 1–7 hours, due to variation in uptake rates and limitations of worker speed. This pulse period is comparable to that used in a similar study by Philip et al. (2011). Carbon dioxide concentrations within the bags were measured at the beginning and end of the pulse using a LI-6251 CO_2_ analyzer (LI-COR Biosciences, Lincoln, NB). In most cases, the seedlings had consumed all of the CO_2_. At the end of the pulse, each root box was moved to the outside of the growth chamber and the labeling bag was then removed to minimize cross contamination. Root boxes were then placed back in their respective growth chambers for a 10–13 day chase period to maintain the treatment conditions prior to harvest. Unlabeled donor seedlings associated with dead receiver seedlings and growing in close proximity to labeled donors were selected for isotope analysis to determine potential uptake of ^13^CO_2_ respired by labeled seedlings (aerial contamination).

Just prior to the commencement of labeling seedlings with ^13^CO_2_, all growth chamber reservoirs were emptied and refilled with water enriched with deuterium (D) at a level of 16.810‰δD (Spectra Stable Isotopes, Columbia, MD). Donor seedlings were continuously labeled with deuterated water (D_2_O) for 10–18 days prior to being harvested in each trial. Reservoirs were refilled to capacity daily with water of the same enrichment level.

### Seedling measurements

Donor and receiver seedlings were growing under the same conditions throughout the experiment, except that donor seedling taproots had access to the reservoir. After receiver seedlings had grown for 1 month, all treatment factors were applied and each seedling was monitored for survival three times per week until harvest (February 2008 for run 1 and February 2009 for run 2). Once treatments had commenced for a month, seedlings were measured for chlorophyll fluorescence. Seedling leaves were dark adapted for a minimum of 20 min prior to measurement with a chlorophyll fluorometer OS-30p (Opti-Sciences, Hudson, NH) (see Equation A1 for calculation).

Growth of surviving seedlings was measured by oven-DW (65°C for 48 hours) of root and shoot biomass. Prior to drying, weighing, and milling, the xylem of pulse-labeled donor seedlings and their associated receiver seedlings was removed at harvest and frozen until the water was extracted following Schoonmaker et al. (2007). After water extraction, the xylem of each seedling was reunited with the remainder of the shoot, and both shoots and roots were milled. Milled biomass samples were analyzed for δ^13^C by mass spectrometry using a PDZ Europa (Sercon Ltd., Cheshire, UK), and xylem water was analyzed for δD and δ^18^O by tunable diode laser using a LGR DLT-100 (Los Gatos Research, Inc., Mountain View, CA) at the University of California Davis Stable Isotope Facility (UCDSIF) (see Equation A2 for calculation). This was done to assess transfer of C and H_2_O from the donor to the receiver seedling, as well as evaporative enrichment of the water.

δD was converted into percent of xylem water originating from reservoir following procedures outlined in Schoonmaker et al. (2007). The xylem water extracted for each treatment combination was of insufficient quantity for UCDSIF measurement protocol; thus, we pooled samples across soil moisture and temperature treatments, because the mesh and [CO_2_] factors were the most novel and costly of this study.

### Data analysis

Receiver seedlings that were growing with a dead donor were excluded from the analysis. Effects of the treatments on receiver seedling survival, growth, δ^13^C, percent of xylem water originating from the reservoir, and F_v_/F_m_ were analyzed using the SAS System for Windows, V9.2 (2009). Logistic regression analysis was used to determine whether seedling survival was associated with mesh treatment, soil moisture regime, temperature regime, CO_2_ regime, or PAR (SAS PROC LOGISTIC) (Tabachnick and Fidell 2001) (see Equation A3 for model form).

The predictive factors were allowed to enter the model if they improved the overall fit, but were ultimately removed from the model if they did not meet the criteria of*P*≤ 0.05, with the stipulation that the treatment factors and interactions had to be retained until all remaining covariates were significant.

All growth, isotope, and fluorescence analyses were performed as an analysis of covariance (ANCOVA) for a factorial set of treatments using PAR, δ^18^O of donor seedling xylem water, δ^18^O of receiver seedling xylem water, percent reservoir water taken up by donor seedlings, percent reservoir water taken up by receiver seedlings, postlabeling chase period (for δ^13^C), labeling period (for δD), and trial run as covariates in a completely randomized design using SAS PROC MIXED (Milliken and Johnson 2002) (see Equation A4 for model form).

The procedure for entry and retention of the covariates was the same as that of the survival analysis, except that the requirement for retention of covariates was*P*≤ 0.1, and treatment factors and interactions were retained regardless of*P*-value. Growth and δ^13^C were logarithmically transformed, while F_v_/F_m_ was power transformed, to conform to the assumptions of ANCOVA.

## Results

A subsample of donor and receiver seedlings was visually examined for colonization after harvest, and we found that 94% of root tips were colonized. The same EM fungal taxa occurred on both the donor and the receiver seedlings, and included*Wilcoxina rehmii, Rhizopogon/Suillus, Cenococcum geophilum, DSE, Amphinema byssoides*, and an unknown type. The molecular identities of these EM fungal taxa are reported in Bingham and Simard (2011). In a companion field study, old trees and seedlings shared eight EM fungal taxa over 60% of their root tips, indicating strong networking potential (Bingham and Simard 2011).

**Figure 3:**
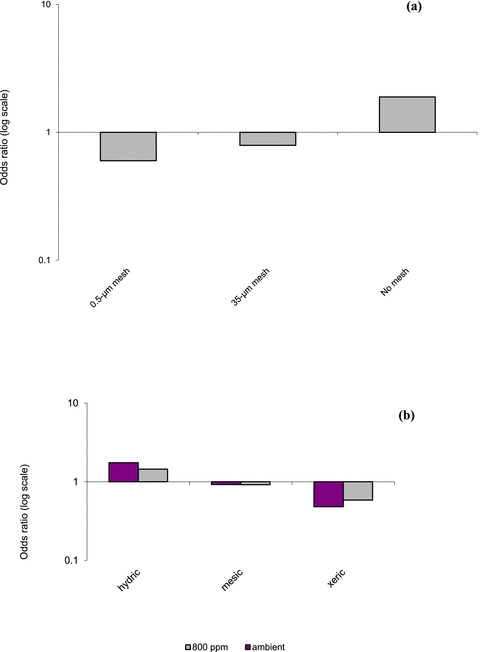
Odds ratio values (logarithmic y-axis scale) for (A) mesh treatment and (B) the interaction of [CO_2_] and soil moisture in the logistic regression model predicting survival of receiver seedlings. The odds ratio for a category is the odds of survival of a seedling in that particular category relative to seedlings not in that category, after adjusting for covariates.

Of the 624 receiver seedlings that were retained in the analysis, 360 survived to harvest. The logistic model predicting receiver seedling survival included [CO_2_], soil moisture regime, mesh treatment, and the interaction of [CO_2_] with soil moisture regime as significant predictors (Wald χ^2^ = 150.7, df = 8,*P* < 0.0001,*c* = 0.842) (Table 1). PAR also entered the model as a covariate. None of the other variables and interactions tested entered the model at*P* < 0.05. The probability of survival was lowest among receiver seedlings planted in 0.5-µm mesh, and greatest among seedlings growing in no mesh (Fig. 3A). Contrary to expectations, receiver seedling survival decreased under CO_2_ enrichment. Among the soil moisture treatments, the probability of survival was lowest in the xeric and highest in the hygric treatment (Fig. 3B). As expected, the receiver seedlings growing in xeric soil performed best when growing at 800 ppm CO_2_. Unexpectedly, the receiver seedlings growing in hygric soil had the poorest performance when growing at 800 ppm CO_2_. Additionally, the probability of survival decreased by 2% for every 1-μmolphoton/m^2^/sec increase in PAR.

**Table 1:** Logistic regression testing for the probability of seedling survival in response to, [CO_2_] (ppm), mesh treatments, soil moisture regime, temperature regime, and PAR (µmol/m^2^/sec). Temperature was removed from the original model

Logistic regression:*c* = 0.842		Likelihood ratio*P* < 0.0001		
	
Effect	Odds ratios	df	Wald χ^2^	*P*> χ^2^
800 ppm CO_2_	0.970516	1	5.7755	0.0163
Ambient CO_2_	1.03038			
Xeric	0.479438	2	103.6506	<0.0001
Mesic	0.901919			
Hygric	1.908867			
0.5-µm mesh	0.6	2	89.5458	<0.0001
35-µm mesh	0.791281			
No mesh	1.891892			
800 ppm ×xeric	0.585091	2	5.9129	0.052
800 ppm ×mesic	0.917258			
800 ppm ×hygric	1.447368			
Ambient ×xeric	0.48221			
Ambient ×mesic	0.921053			
Ambient ×hygric	1.747814			
PAR	0.983635	1	7.4978	0.0062

**Figure 4:**
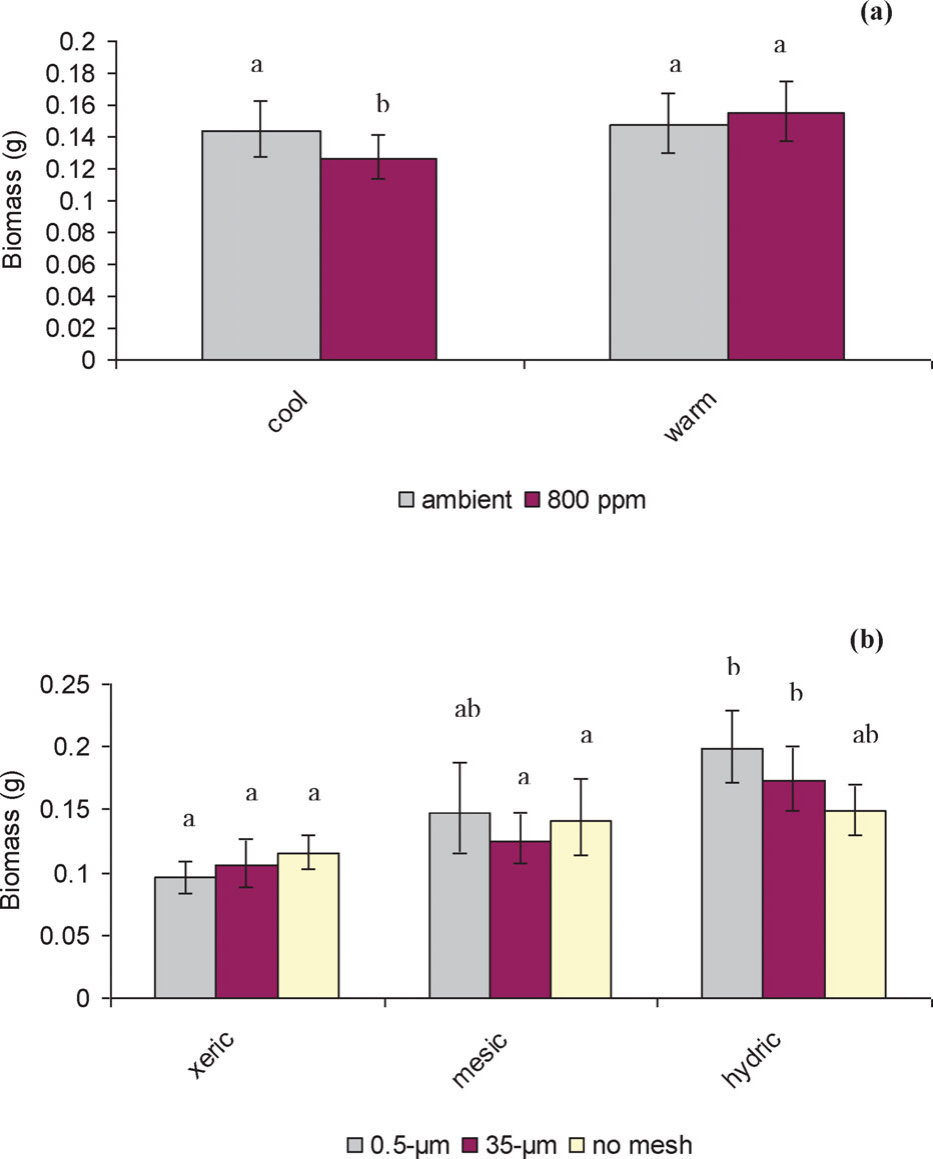
Unadjusted means for total receiver seedling biomass under (A) various combinations of temperature regime and [CO_2_] and (B) various combinations of soil moisture regime and mesh treatments. Bars with different letters are significantly different at*P*≤ 0.05. Error bars are 1 SE.

Growth of receiver seedlings was affected by [CO_2_] (*P* = 0.0094), temperature (*P* = 0.0017), soil moisture (*P* < 0.0001), and the interaction between [CO_2_] and temperature regime (*P* = 0.0448), as well as the interaction between soil moisture regime and mesh treatment (*P* = 0.0475), when PAR was included as a covariate (Table 2). Growth differences between the warm and cool temperature regimes were greater under the high than under the low [CO_2_] (Fig. 4A). Growth was highest under warm temperatures at both CO_2_ levels and lowest under cool temperatures at 800 ppm CO_2_. While growth increased with soil moisture, this effect was mediated by the mesh treatments, with growth exhibiting a general decrease with increasing pore size under hygric conditions (i.e., greatest in the 0.5-µm mesh and lowest in no mesh), and no effect of pore size under mesic or xeric conditions (Fig. 4B). Growth decreased with PAR, and this response differed in magnitude by run.

**Table 2:** Analysis of covariance testing for response of the natural logarithm of total biomass of seedlings to [CO_2_] (ppm), temperature regime, soil moisture regime, and mesh treatment after adjustment for PAR (µmol/m^2^/s) nested within run

ANCOVA: AIC = 238.4		

Effect	F-value	*P* > F
[CO_2_]	6.9	0.0094
Temperature	10.16	0.0017
[CO_2_] × Temperature	4.09	0.0448
Soil moisture	22.97	<.0001
[CO_2_] × Soil moisture	0.19	0.8286
Temperature × Soil moisture	0.13	0.8762
[CO_2_] × Temperature × Soil moisture	0.85	0.4279
Mesh	0.16	0.8537
[CO_2_] × Mesh	0.69	0.5019
Temperature × Mesh	0.53	0.5921
[CO_2_] × Temperature × Mesh	0.75	0.4736
Soil moisture × Mesh	2.46	0.0475
[CO_2_] × Soil moisture × Mesh	0.21	0.9353
Temperature × Soil moisture × Mesh	0.12	0.9765
[CO_2_] × Temperature × Soil moisture × Mesh	1.13	0.3397
Covariates		
PAR (2007–2008) (µmol/m^2^/s)	−3.61	0.0004
PAR (2008–2009) (µmol/m^2^/s)	−3.71	0.0003

Note. The coefficient signs are only given for continuous variables.

**Figure 5:**
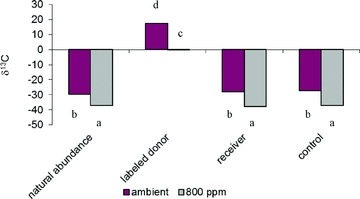
Means for δ^13^C of seedlings among natural abundance controls, labeled donors, receivers, and postlabel controls. Error bars are 1 SE.

The δ^13^C of labeled donor seedlings averaged 8.64‰, while natural abundance, receiver, and chase period-control seedlings were statistically indistinguishable, with an average δ^13^C of –32.8‰ (Fig. 5). The δ^13^C of receiver seedlings was affected by [CO_2_] (*P*≤ 0.0001), and was higher under ambient CO_2_ than 800 ppm CO_2_. The same was true for natural abundance and control seedlings.

**Figure 6:**
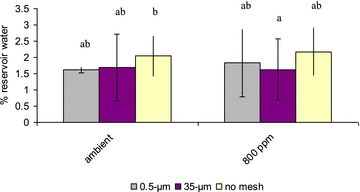
Unadjusted means for the percent reservoir water taken up by receiver seedlings (calculated from δD) under various combinations of [CO_2_] and mesh treatments. Bars with different letters are significantly different at*P*≤ 0.1. Error bars are 1 SE.

Because of the reduced replication that resulted from pooling samples, we relaxed the threshold for significance of the main effects to*P*≤ 0.1.The δD of receiver seedlings was affected by mesh treatment (*P* = 0.0298), (CO_2_) (*P* = 0.0735), and the interaction of mesh treatment and [CO_2_] (*P* = 0.0596), when δ^18^O of donor seedling xylem water, δ^18^O of receiver seedling xylem water, percent reservoir water taken up by donor seedlings, labeling period, and trial year were included as covariates (Table 3). δD of receiver seedling xylem was highest when they were growing with no mesh under ambient CO_2_ and lowest in 35-µm mesh at 800 ppm [CO_2_] (Fig. 6). The δD of receiver seedlings decreased with δ^18^O and δD of donor seedlings, and increased with δ^18^O of receiver seedling xylem water and postlabeling chase period.

**Table 3:** Analysis of covariance testing for response of the percent reservoir D_2_O taken up by receiver seedlings to mesh treatments and [CO_2_] (ppm), after adjustment for δ^18^O of donor seedling xylem water, δ^18^O of receiver seedling xylem water, percent reservoir water taken up by donor seedlings, labeling period, and run

ANCOVA: AIC = 11.8			

Effect	Coefficient	F-value	*P*> F
Mesh	N/A	563.8	0.0298
[CO_2_]	N/A	74.33	0.0735
Mesh × [CO_2_]	N/A	140.37	0.0596
Covariates			
Donor δ^18^O (‰)	-	208.91	0.044
Donor % reservoir uptake	-	76.73	0.0724
Receiver δ^18^O (‰)	+	1143.67	0.0188
Labeling period (days)	+	149.42	0.052

Note. The coefficient signs are only given for continuous variables.

Maximum quantum yield of photosystem II (Fv/Fm) of receiver seedlings was affected by soil moisture (*P* = 0.0219), the interaction between [CO_2_] and temperature regime (*P* = 0.0206), and the interaction among [CO_2_], temperature regime, and mesh treatment (*P* = 0.036) (Table A1). Fluorescence was highest when seedlings grew in 0.5-µm mesh under cool temperatures and low [CO_2_], and lowest when seedlings grew in the 35-µm mesh under cool temperatures and high [CO_2_] (Fig. A1). Those growing in the 35-µm mesh under cool temperature and high [CO_2_] also exhibited the highest variability of any treatment combination. Fluorescence increased with soil moisture.

## Discussion

Regardless of environmental treatment, survival of receiver seedlings increased as the potential to form MNs with donor seedlings increased; it was highest where there was no mesh and lowest in 0.5-µm mesh. Survival was also affected by an interaction between [CO_2_] and soil moisture regime, such that it was maximized under elevated [CO_2_] in xeric soil conditions and minimized under elevated [CO_2_] in hygric soil conditions. Overall, these results are consistent with our first hypothesis that MNs would facilitate seedling establishment and our expectation that increased [CO_2_] would alleviate water deficiency in the driest soils. The mesh factor did not interact with water-deficiency related factors as hypothesized, however; regardless of soil moisture regime, seedling survival was increased when able to form an MN. We are confident that seedlings were limited by water availability in the driest treatment, and thus MNs would have alleviated this limitation. By contrast, seedlings were likely limited by N in the wettest treatment, because seedlings were clearly chlorotic, and we did not fertilize because N amendment is known to reduce EM mycelial growth (Arnebrant 1994). If seedlings in the wettest environments were severely N-limited, they might benefit from a larger mycelium associated with the MN through uptake from a greater soil volume or plant-to-plant N-transfer (He et al. 2003) early in the experiment, before competition with the donor became prevalent. However, under both water and N limitation, seedlings would have had direct access to the greatest volume of soil in the no mesh treatments. We think the reduction in survival with PAR was probably a combination of increased rates of soil drying combined with increased photooxidative stress for already weakened seedlings.

MNs acted independently of other treatment factors to affect survival, whereas they interacted with soil moisture regime to affect growth. The general pattern, that mean total biomass did not change with access to MNs or roots under xeric conditions while declining with increasing network access in the highest soil moisture treatment, was generally consistent with our second hypothesis, that seedling growth would benefit least from MNs under water sufficient conditions. This pattern may simply be driven by N competition between the seedlings rather than the presence of MNs. Because root and hyphal growth were unrestricted in the absence of mesh, and hyphal growth was unrestricted in the 35-µm mesh, we cannot rule out that a mechanism for the mesh effects observed in any of our results is freedom of root and hyphal exploration independent of formation of a continuous hyphal connection between seedling root tips. This mechanism would comprise both increased volume of soil accessible to the receiver, as well as the potential for increased root competition with the donor. Unexpectedly, high [CO_2_] reduced growth under cool temperatures, resonating with the reduction in survival associated with high [CO_2_] under the hygric moisture level. The explanation that seems most plausible to us is that soil stayed moist for longer periods of time in both the hygric moisture level and cool temperature regime because seedlings were not transpiring as rapidly under high [CO_2_], and these conditions were more conducive to weakening seedlings by N deficiency or soil-borne pathogens. While we made every effort to keep conditions in each trial identical, there were bound to be some small differences, and this included differential drifting in PAR between trials, which we believe accounts for the difference in magnitude of the decrease in growth with PAR between the two trials.

δ^13^C did not increase with network forming potential, leading us to reject part of our third hypothesis, that plant-to-plant C transfer was one mechanism by which MNs affected seedling survival and growth. Although ^13^C enrichment was unaffected by MN potential, it was affected by [CO_2_], where receiver seedling δ^13^C in ambient [CO_2_] was close to 10‰ higher that in elevated [CO_2_]. Carbon dioxide respired by donor roots should comprise a greater proportion of available CO_2_ in the chamber under the ambient [CO_2_] treatment than it does under the 800 ppm [CO_2_] treatment. However, the difference observed in natural abundance, labeled donor, receiver, and control seedlings in δ^13^C between ambient and 800ppm [CO_2_] treatments (Fig. 6), is easily accountable for by the difference in δ^13^C of ambient CO_2_ (averaged approximately –8.2‰ during the experiment) and fossil fuel derived CO_2_ (approximately -44‰) used for enrichment. While this effect is not surprising, we also expected δ^13^C to differ by mesh treatment, with network potential increasing C transfer. Severe N deficiency in this system may have masked network effects because most C is likely transferred through MNs with N in amino acids (e.g., glutamine) (He et al. 2003).

In contrast to C, transfer of water was facilitated in the no mesh treatment when seedlings were growing under ambient [CO_2_]. Here, the xylem δD of receiver seedlings was affected by the interaction between mesh and [CO_2_] factors. The xylem δD tended to be greatest for receiver seedlings growing in no mesh under ambient [CO_2_]. This could be explained by simple hydraulic redistribution, however more numerous and close contact root and hyphal network connections with the donor in the no mesh treatment could have facilitated this redistribution. At 800 ppm [CO_2_], however, δD of receiver seedlings tended to be lowest in the 35-µm mesh, suggesting that water movement to receivers was occurring mainly via the soil matrix, and that MNs were unimportant in transferring water at elevated [CO_2_]. The δ^18^O values not only relate to variation in δD through evaporative fractionation at the stomata, but also to variation in uptake of reservoir water that is less enriched in ^18^O than water that has become evaporatively enriched at the soil surface, when soil moisture is depleted and seedlings are transpiring more at higher temperatures. Therefore, using it as a covariate captures more of the variation in δD, and helps to capture some of the variation in δD originating from the temperature and soil moisture treatments, both of which had to be removed as factors for the pooling of xylem water.

Our study showed that interior Douglas-fir seedling regeneration parameters in laboratory conditions were affected by temperature regime, [CO_2_], and the interaction of soil moisture regime with MN potential, but the potential MN effects did not appear to be related to C transfer, and were only weakly related to water transfer. We acknowledge that access to a greater volume of soil via hyphae and roots in the no mesh treatment could explain some of these patterns. We conclude, where water-deficient seedlings were growing near well-hydrated seedlings, that: (1) survival of receiver seedlings increased with MN potential, as well as the interaction of [CO_2_] and soil moisture regime, with elevated [CO_2_] improving seedling survival under soil moisture deficiency; (2) growth of receiver seedlings increased with MN potential under xeric, but not hygric conditions; (3) growth of receivers also declined, however, when temperature was low and [CO2] was high; (4) C transfer to receiver seedlings was not affected by MN potential; (5) hydraulic redistribution to receiver seedlings increased with MN potential under ambient [CO2], but the overall pattern was complex, and the data were not sufficient to resolve the precise mechanism for this; and (6) chlorophyll fluorescence of receiver seedlings was reduced with MN potential under low temperature and high [CO_2_], again presenting a complex overall pattern. Thus, the results of this experiment are consistent with the hypothesis that MNs are important to interior Douglas-fir seedling establishment and growth under water-deficient conditions; however, we cannot exclude uninhibited root and/or hyphal exploration as the driver of these patterns. Moreover, the data also partially support the hypothesis that water transfer plays a role in this facilitation, but that C transfer appears to be unimportant. EM networks may increase in importance for forest regeneration where climate change increases water stress.
